# Gender differences in the association between sleep duration and body mass index, percentage of body fat and visceral fat area among chinese adults: a cross-sectional study

**DOI:** 10.1186/s12902-021-00913-4

**Published:** 2021-12-24

**Authors:** Yaqi Fan, Li Zhang, Yuxue Wang, Chunjun Li, Binbin Zhang, Jiangshan He, Pei Guo, Xin Qi, Mianzhi Zhang, Congfang Guo, Yirui Guo, Minying Zhang

**Affiliations:** 1 School of Medicine, Nankai University, 94, Weijin Road, 300071 Tianjin, China; 2 Tianjin First Central Hospital, Tianjin, China; 3 Tianjin People’s Hospital, Tianjin, China; 4 Dongfang Hospital, Beijing University of Chinese Medicine, Beijing, China; 5 Tianjin Academy of Traditional Chinese Medicine Affiliated Hospital, Tianjin, China

**Keywords:** Sleep duration, Body mass index, Percentage of body fat, Visceral fat area, Gender difference

## Abstract

**Background:**

The relationship between sleep duration and anthropometric indices are still unclear. This study aimed to explore the association between sleep duration and body mass index (BMI), percentage of body fat (PBF) and visceral fat area (VFA) among Chinese adults, further to explore gender difference in it.

**Methods:**

We analyzed part of the baseline data of a cohort study among adult attendees at two health-screening centers in China. Sleep duration was self-reported and categorized into short (< 7 h/day), optimal (7-9 h/day) and long sleep (≥ 9 h/day). BMI, PBF and VFA were assessed by bioelectric impedance analysis. Demographic characteristics, chronic diseases and medication history, physical activity, smoking and alcohol drinking behaviors were measured by an investigator-administrated questionnaire.

**Results:**

A total of 9059 adult participants (63.08% were females) were included in the analysis. The participants aged from 19 to 91 years with the mean age of 45.0 ± 14.6 years. Short sleep was independently associated with elevated odds of general obesity (defined using BMI) and visceral obesity (defined using VFA) among the total study population, and gender differences were observed in these associations. Among women, short sleep was associated with 62% increased odds of general obesity (OR = 1.62, 95% CI: 1.24-2.12) and 22% increased odds of visceral obesity (OR = 1.22, 95% CI: 1.02-1.45). Among men, long sleep duration was associated with 21% decreased odds of visceral obesity (OR = 0.79, 95% CI: 0.64-0.99). No association was observed between sleep duration and PBF in both sexes.

**Conclusions:**

Sleep duration was associated with increased odds of general and visceral obesity, and this association differed between men and women. No association was observed between sleep duration and PBF among either males or females.

**Supplementary Information:**

The online version contains supplementary material available at 10.1186/s12902-021-00913-4.

## Background

Obesity has become one of the most serious public health problems globally [1]. In parallel to the elevated prevalence of obesity, sleep deprivation and poor sleep quality are also increasing globally [2–6]. The findings on the relationship between sleep duration and obesity remain controversial. Though the evidence for sleep deprivation as a risk factor for obesity is strong [7–10], a cohort study reported no significant association between sleep duration and obesity [11]. In addition, serval studies reported inconsistent gender differences in this association, for example, Ko et al. found an inverse trend between sleep duration and BMI only in men (r = -0.054, *p* = 0.011) [12]; whereas a Taiwanese study indicated an opposite result that women with < 6 h sleep per day exhibited an increased likelihood of general obesity (OR= 3.16, 95% CI: 1.33-7.53).

BMI is the most widely used indicator to assess obesity because it is the easiest to measure. Evidence shows that BMI has high specificity but low sensitivity in identifying obesity, approximately 50% of individuals with excess body fat percent are labeled as non-obese by BMI [13]. BMI could identify if a person is overweight, but could not distinguish between fat and muscle. Individuals who have a normal BMI but high body fat are more likely to have metabolic dysregulation compared with those with normal weight and low fat mass [13], whereas PBF can explain the percentage of body fat content. Excess visceral adipose tissue accumulation is referred to as visceral obesity, which may be defined by VFA. Visceral obesity is proven to be associated with elevated prevalence of metabolic syndrome and cardiovascular diseases [14–16].

Up to now, the results of the correlation between sleep duration and PBF are inconsistent. Several studies have suggested that there may be no relationship between sleep duration and PBF [17, 18]; however, this was contrary to the Swedish study [19] which found that both habitual short and long sleep were associated with higher fat mass. In addition, gender differences in this correlation also have mixed results [20, 21]. As for the association between sleep duration and visceral fat area, only a few studies have analyzed it. According to a study in South Korea [22], compared with > 7 h of sleep, sleep < 5 h was associated with elevated odds of visceral obesity; but Yi et al. found that sleep duration was not associated with VFA whether the covariates were adjusted or not [23].

Studies to date have been inconsistent in the relationship between sleep duration and different obesity indicators. Additionally, gender differences are still unclear. We analyzed the baseline data of a cohort study in Beijing-Tianjin-Hebei Region, China, to investigate the associations between self-report sleep duration and BMI, PBF, and VFA; further explore whether there are gender differences in the associations found.

## Methods

### Study participants

This study analyzed part of the baseline data of the Cohort Study among General Population in the Beijing-Tianjin-Hebei Region, a National Key R&D Program of China. The participants were recruited by cluster random sampling among attendees at two health-screening centers in Tianjin from August 2018 to December 2019. Attendees met following criteria were included in the study: 18 years or older; voluntarily participated in the survey and signed informed consent. Exclusion criteria were: with cognitive impairment, hearing or language impairment, having mental disease, with pacemakers installed, disabled, taking weight-loss drugs. The study protocol was approved by the ethics review boards of Nankai University, Tianjin First Central Hospital and Tianjin People’s Hospital where the survey was conducted. Written informed consent was obtained from all participants.

### Outcome variable

According to Chinese guidelines [24, 25], general obesity was defined as BMI ≥ 28 kg/m^2^; high PBF was defined as PBF > 25% in men and > 35% in women [26]; and visceral obesity was defined as VFA ≥ 100 cm^2^ [27]. Height was measured to the nearest 0.1 (cm) by the professional medical staff of the medical examination centers using the same device (GL-310, Seoul, Korea). BMI, PBF and VFA were estimated by bioelectrical impedance analysis (BIA, InBody 770, Biospace, Seoul, Korea). The age, sex and height of participants were input into the system prior to the assessment.

### Independent variables

The simplified Pittsburgh Sleep Quality Index (PSQI) was used to assess sleep duration with the questions, “During the past month, what time have you usually gone to bed at night?”, “During the past month, how long has it usually taken you to fall asleep each night?” and “During the past month, what time have you usually gotten up in the morning?”. Based on the responses, we calculated the participants’ average daily night sleep duration. According to the National Sleep Foundation, the optimal sleep duration for adults is 7-9 h per night [28]. However, few participants reported to sleep > 9 h in this study, so we categorized sleep duration into three groups, < 7 h, 7-9 h (including 7 h but not including 9 h) and ≥ 9 h.

### Covariates

Using the questionnaire, we also collected demographic characteristics including age, occupation (civil servants, professionals or other), marriage status (unmarried, married, divorced or widowed), education level (high school and below, bachelor degree or postgraduate and above); history of chronic diseases (yes, no or unknown) including hypertension, diabetes, coronary heart disease, stroke, chronic kidney disease, chronic obstructive pulmonary disease, and dyslipidemia, cancer, and individuals suffering from one of them is define as with chronic diseases; taking medicine regularly in prior three months (lowering uric acid agents, anti-arrhythmia agents, hormone agents and sleeping agents); behaviors including smoking (yes, never or former), alcohol drinking (yes, never or former), exercise (never, occasional or regular). Participants exercising less than once a week was labeled as never, a total of less than an hour of exercise per week was marked as occasional. Subjective sleep quality was asked by the following question: “During the past month, how would you rate your sleep quality overall?” The options were categorized into four groups: very good, good, bad, very bad. Tsai et al. [29] proposed that subjective sleep quality had the highest correlation with the global score among the seven factors when they evaluated the Chinese version of the Pittsburgh Sleep Quality Index (PSQI). So, we only used subjective sleep quality as the indicator of sleep quality. The covariates were all self-reported.

### Statistical analyses

The questionnaire information was double input using Epidata (version 3.0) software, and all the analyses were performed using SPSS (version 25.0) software. The participants were grouped according to sleep duration, measurement data consistent with normal distribution were expressed as mean ± s.d., one-way analysis of variance (ANOVA) was used for comparison between groups; enumeration data were presented as n%, Chi-square test was used for comparison between groups. Logistic regression models were constructed to evaluate the adjusted associations between sleep duration and general obesity, high PBF and visceral obesity. In the logistic regression analysis, we converted continuous variables to rank variables, age was divided into three groups: < 30, 30~59 and ≥ 60 years. Model 1 was adjusted for potential confounding factors including age, occupation, marriage status, education level, smoking, alcohol drinking and exercise; In addition to potential confounding factors of Model 1, we adjusted for subjective sleep quality in Model 2; In additional to potential confounding factors of Model 2, history of chronic diseases and regular medication in prior three months were adjusted in Model 3. Two-side *P* < 0.05 was considered statistically significant. In order to investigate the difference between men and women, we also conducted sex subgroup analysis.

## Results

A total of 9,509 adults including 5,714 females and 3,795 males were included in the analysis, the age ranged from 19 to 91 years with the mean age of 45.0 ± 14.6 years. Overall, 12.7% of women and 13.7% of men reported sleeping < 7 h, 75.4% of women and 75.5% of men reported sleeping 7-9 h, and 11.9% of women and 10.8% of men reported sleeping ≥ 9 h. Table 1(Table 1 was placed at the end of the document text file) shows the demographic characteristics and behavioral factors of the participants according to sleep duration. Those who reported sleeping < 7 h were older and more likely smokers, reported less exercise, poorer sleep quality and had higher prevalence of chronic diseases. Participants who were divorced or widowed were also more likely to be sleep-deprived (all *P* < 0.05). Optimal sleepers had higher education, seldom smoked or drank, exercised more and were less likely to suffer from chronic diseases (all *P* < 0.05).

**Table 1:** The demographic characteristics and behavioral habits of the participants according to sleep duration (*n* = 9509)

Variables	Sleep Duration			*P*
	< 7 h	7-9 h	≥ 9 h	
Age	46.76 ± 14.547	44.83 ± 14.495	44.38 ± 15.158	< 0.001
Gender				0.074
Women	723 (12.7%)	4308 (75.4%)	683 (11.9%)	
Men	520 (13.7%)	2866 (75.5%)	409 (10.8%)	
Occupation				0.291
Civil servants	333 (12.3%)	2050 (75.6%)	329 (12.1%)	
Professionals	749 (13.6%)	4135 (75.3%)	610 (11.1%)	
Other	161 (12.4%)	989 (75.9%)	513 (11.7%)	
Marriage status				0.137
Unmarried	143 (11.5%)	943 (75.9%)	156 (12.6%)	
Married	1067 (13.2%)	6095 (75.5%)	915 (11.3%)	
Divorced/Widowed	33 (17.4%)	136 (71.6%)	21 (11.1%)	
Education level				0.245
High school and below	119 (13.0%)	680 (74.5%)	114 (12.5%)	
Bachelor degree	752 (13.3%)	4251 (74.9%)	672 (11.8%)	
Postgraduate and above	372 (12.7%)	2243 (76.8%)	306 (10.5%)	
Smoking				< 0.001
Yes	209 (19.9%)	757 (72.0%)	86 (8.2%)	
Never	975 (12.0%)	6170 (76.0%)	969 (11.9%)	
Former	59 (17.3%)	246 (71.9%)	37 (10.8%)	
Alcohol drinking				0.002
Yes	230 (15.1%)	1118 (73.5%)	174 (11.4%)	
Never	989 (12.5%)	5988 (76.0%)	905 (11.5%)	
Former	24 (22.9%)	68 (64.8%)	13 (12.4%)	
Exercise				< 0.001
Never	523 (15.3%)	2552 (74.6%)	348 (10.2%)	
Occasional	373 (10.9%)	2565 (75.2%)	475 (13.9%)	
Regular	347 (13.0%)	2057 (77.0%)	269 (10.1%)	
Subjective sleep quality				< 0.001
Very Good	375 (10.9%)	2624 (76.5%)	432 (12.6%)	
Good	619 (12.9%)	3643 (75.9%)	540 (11.2%)	
Bad	223 (19.4%)	814 (70.9%)	111 (9.7%)	
Very Bad	26 (20.3%)	93 (72.7%)	9 (7.0%)	
Chronic diseases				0.005
Yes	332 (15.5%)	1565 (73.0%)	248 (11.6%)	
No	907 (12.4%)	5591 (76.2%)	842 (11.5%)	
Unknowing	4 (16.7%)	18 (75.0%)	2 (8.3%)	
Cancer				0.111
Yes	12 (19.7%)	45 (73.8%)	4 (6.6%)	
No	1230 (13.0%)	7120 (75.5%)	1084 (11.5%)	
Unknowing	1 (7.1%)	9 (64.3%)	4 (28.6%)	
Regular medication in last three months				0.008
Yes	127 (16.7%)	549 (72.1%)	85 (11.2%)	
No	1116 (12.8%)	6625 (75.7%)	1007 (11.5%)	

Table 2 shows the mean BMI, PBF and VFA of the participants according to sleep duration. The differences in BMI and VFA between short, optimal and long sleepers were statistically significant, but that in PBF were not significant. The results of sex subgroup analyses showed that there were significant differences in both BMI and VFA between short, optimal and long sleepers (*P* < 0.05), but not in PBF (*P* > 0.05) among women. Among men, no statistically significant differences in BMI, PBF or VFA were found between short or long and optimal sleepers (*P* > 0.05).

**Table 2:** The BMI, PBF and VFA of the participants according to sleep duration (*n*=9509)

Variables	Sleep Duration			*P*
	< 7 h	7-9 h	≥ 9 h	
All (*n*=9509)	1243	7174	1092	
BMI(kg/m²)	24.38 ± 3.52	24.08 ± 3.45	24.00 ± 3.45	0.011
PBF(%)	29.76 ± 6.89	29.65 ± 6.67	30.06 ± 6.67	0.161
VFA(cm²)	95.54 ± 35.46	92.42 ± 34.15	92.33 ± 35.07	0.011
Women (*n*=5714)	723	4308	683	
BMI(kg/m²)	23.44 ± 3.47	23.09 ± 3.21	23.11 ± 3.24	0.030
PBF(%)	32.43 ± 6.43	32.09 ± 6.00	32.42 ± 5.99	0.197
VFA(cm²)	93.90 ± 37.70	89.86 ± 34.62	90.86 ± 35.85	0.016
Men (*n*=3795)	520	2866	409	
BMI(kg/m²)	25.68 ± 3.15	25.57 ± 3.25	25.47 ± 3.28	0.611
PBF(%)	26.06 ± 5.68	25.98 ± 5.89	26.11 ± 5.83	0.881
VFA(cm²)	97.82 ± 31.97	96.26 ± 33.08	94.78 ± 33.61	0.373

Logistic regression showed that, after adjusting for the covariates, short sleep was associated with elevated odds of general obesity (OR = 1.22, 95% CI: 1.02-1.45) and visceral obesity (OR = 1.14, 95% CI: 1.01-1.30), but not with high PBF; whereas long sleep was not associated with general obesity, high PBF or visceral obesity (Table 3).

**Table 3:** The OR of general obesity, high PBF and visceral obesity among the total of participants according to sleep duration (*n*=9509)

Variables	Model	OR (95% CI)		
		Sleep Duration		
		< 7 h	7-9 h	≥ 9 h
General obesity	Model 1	1.22 (1.02-1.44)	reference	0.90 (0.74-1.10)
	Model 2	1.24 (1.04-1.47)	reference	0.90 (0.73-1.09)
	Model 3	1.22 (1.02-1.45)	reference	0.88 (0.72-1.08)
High PBF	Model 1	1.04 (0.92-1.17)	reference	0.99 (0.86-1.13)
	Model 2	1.03 (0.91-1.17)	reference	0.99 (0.86-1.13)
	Model 3	1.01 (0.89-1.15)	reference	0.98 (0.87-1.12)
Visceral obesity	Model 1	1.17 (1.03-1.33)	reference	0.96 (0.84-1.11)
	Model 2	1.17 (1.02-1.33)	reference	0.96 (0.84-1.11)
	Model 3	1.14 (1.01-1.30)	reference	0.95 (0.83-1.10)

Model 1 was adjusted for age, occupation, marriage status, education level, smoking, alcohol drinking and exercise; Model 2 was adjusted for subjective sleep quality in addition to Model 1; Model 3 was adjusted for chronic diseases, cancer and regular medication in last three months in addition to Model 2

Sex subgroup analysis indicates that, after completely adjusting for the covariates, sleep deprivation was independently associated with increased odds of general obesity (OR = 1.62, 95% CI: 1.24-2.12) and visceral obesity (OR = 1.22, 95 CI: 1.02-1.45) in women (Table 4). However, in men, long sleep was independently associated with decreased odds of visceral obesity (OR = 0.79, 95 CI: 0.64-0.99), but neither short nor long sleep was associated with general obesity (Table 5). No association between sleep duration and high PBF was found in both sexes.

**Table 4:** The OR of general obesity, high PBF and visceral obesity among women according to sleep duration (*n*=5714)

Variables	Model	OR (95% CI)		
		Sleep Duration		
		< 7 h	7-9 h	≥ 9 h
General obesity				
	Model 1	1.60 (1.23-2.08)	reference	1.08 (0.79-1.48)
	Model 2	1.61 (1.24-2.10)	reference	1.08 (0.79-1.47)
	Model 3	1.62 (1.24-2.12)	reference	1.06 (0.77-1.45)
High PBF				
	Model 1	1.14 (0.96-1.36)	reference	1.05 (0.88-1.26)
	Model 2	1.14 (0.96-1.35)	reference	1.05 (0.88-1.26)
	Model 3	1.13 (0.95-1.35)	reference	1.05 (0.88-1.26)
Visceral obesity				
	Model 1	1.24 (1.04-1.48)	reference	1.09 (0.91-1.30)
	Model 2	1.23 (1.03-1.46)	reference	1.10 (0.92-1.31)
	Model 3	1.22 (1.02-1.45)	reference	1.10 (0.92-1.32)

Model 1 was adjusted for age, occupation, marriage status, education level, smoking, alcohol drinking and exercise; Model 2 was adjusted for subjective sleep quality in addition to Model 1; Model 3 was adjusted for chronic diseases, cancer and regular medication in last three months in addition to Model 2

**Table 5:** The OR of general obesity, high PBF and visceral obesity among men according to sleep duration (*n*=3795)

Variables	Model	OR (95% CI)		
		Sleep Duration		
		< 7 h	7-9 h	≥ 9 h
General obesity				
	Model 1	1.01 (0.80-1.27)	reference	0.82 (0.63-1.08)
	Model 2	1.02 (0.81-1.29)	reference	0.82 (0.63-1.07)
	Model 3	1.03 (0.82-1.30)	reference	0.82 (0.63-1.07)
High PBF				
	Model 1	0.93 (0.76-1.12)	reference	0.94 (0.76-1.16)
	Model 2	0.92 (0.76-1.12)	reference	0.94 (0.76-1.17)
	Model 3	0.92 (0.76-1.11)	reference	0.94 (0.76-1.16)
Visceral obesity				
	Model 1	1.11 (0.91-1.35)	reference	0.80 (0.64-0.99)
	Model 2	1.12 (0.92-1.37)	reference	0.80 (0.64-0.99)
	Model 3	1.11 (0.91-1.35)	reference	0.79 (0.64-0.99)

Model 1 was adjusted for age, occupation, marriage status, education level, smoking, alcohol drinking and exercise; Model 2 was adjusted for subjective sleep quality in addition to Model 1; Model 3 was adjusted for chronic diseases, cancer and regular medication in last three months in addition to Model 2

## Discussion

The current study found that sleep duration was associated with BMI and VFA, and there were gender differences in this relationship. After adjusting for potential confounding factors, short sleep duration was associated with the elevated odds of general obesity and visceral obesity only in women; in men, only long sleep was associated with the decreased odds of visceral obesity. Whereas the association between sleep duration and high PBF was non-significant in both sexes.

The evidence for short sleep as a risk factor for obesity is strong. A large Thai cohort of over 60,000 adults reported that short sleep increased the risk of general obesity in both men and women compared with those slept 7 h [10]. Studies conducted in Spain [30], and Finland [31] showed consistent findings. On the contrary, Mayumi et al. [32] observed a relationship between short sleep and general obesity in men, while no association in women, which was contrast to the current research. Give the large quantitative differences between men (31,477) and women (3770) in this study, we speculate the small sample size of women might not be sufficient to detect the potential association. The association between long sleep and BMI was not observed in our study, however studies in China [33] and Sweden [34] obtained different results that women who sleep longer were more likely to be obese. Some studies found a U-shaped relationship that both short and long sleep durations were associated with obesity [30, 35]. We speculate the difference may be partly caused by culture difference. Studies shows that sleep duration varies across cultures and countries [36, 37], thus alters the associations between sleep profiles and disease risks [38, 39]. Therefore studies on the association between sleep duration and obesity from different countries are needed. Given the lower prevalence of reported short sleep in the current study, the lack of association between short sleep and obesity in men may due to the small number of men reporting short sleep, and future research is needed to further explore the association between sleep duration and obesity.

No significant association between sleep duration and PBF was found in present study. Studies in the United States [17] and China [18] reported the consistent results. However, Chen et al. found an inverse relation between sleep duration and PBF in men but not in women [20]. Another Chinese study [33] indicated that long sleep was positively associated with high PBF in female adults compared with women who slept 7-8 h. The diverse findings may be due to the different cutoffs of sleep duration and statistical methods used in different studies. Yan et al. [33] categorized the sleep duration into 5 categories, < 6 h, 6-7 h, 7-8 h, 8-9 h and ≥ 9 h, with 7-8 h as the reference; Chen et al. [20] used multivariable generalized linear models to investigate the relationship between sleep duration and PBF, in which PBF was logarithmically transformed.

In the current study, short sleep in women and long sleep in men was associated with increased visceral obesity. Kim et al. [29] found that individuals who slept < 5 h had 105% increased odds of visceral obesity compared with those who slept > 7 h [22]. An American study showed that short and long sleep were both associated with increased odds of visceral obesity among persons younger than 40 years [40]. There are also studies reported no association between sleep duration and visceral obesity [23, 41]. The differences between these findings and ours may be explained by the different study populations, sample size, cutoffs used to categorize sleep duration, and the different methods used to measure visceral fat.

Although the exact association between sleep duration and obesity-related indicators is not fully clarified, several potential mechanisms might explain the link between sleep deprivation and obesity. One is by changing the levels of neurohormones that regulate eating behaviors. Acute sleep deprivation leads to a 28% increase in the average level of appetite-stimulating hormones, while the average level of leptin decreases by 18%. Besides, lack of sleep is associated with increases in hunger, appetite, and hedonic food intake, especially for high-calorie food [42]. Another possible way for insufficient sleep to induce obesity is through activation of the sympathetic nervous system. Insufficient sleep can reduce glucose tolerance, increase cortisol levels and heart rate variability. These neuroendocrine changes interfere with the mechanism of blood glucose metabolic regulation, resulting in higher steady-state blood glucose concentration [43]. Additionally, activation of inflammatory pathways may also play a role in the link between short sleep duration and obesity. Studies have confirmed that insufficient sleep is associated with increases in tumor necrosis factor, interleukin 6 and C-reactive protein, which play important roles in obesity [44, 45]. Long sleep causes many similar potential metabolic problems, which may differ from that of short sleep [46, 47]. Several studies have demonstrated that longer sleep duration among older persons can affect energy metabolism and thereby increase the risk of demaged energy homeostasis through various possible complex mechanisms, including poor sleep quality, sedentary lifestyle, unhealthy dietary habits, and circadian rhythm [48]. How dose long sleep influence energy homeostasis among young adults is rarely studied yet. However, in addition to the different study population and design, different cutoffs of long sleep were used may result in different findings.

The strengths of the current study are as follows. First, to our best knowledge, current study was conducted with the largest sample to explore the relationship between sleep duration and PBF in China. Second, previous research on sleep duration and abdominal fat has mostly focused on waist circumference. The study was the first to investigate the relationship between sleep duration and VFA among Chinese adults as far as we know. Third, we adjusted for chronic disease and subjective sleep quality, which may be the causes of self-reported short sleep duration [49], to get more reliable findings on associations between sleep duration and obesity.

Our study also had several limitations. Firstly, the cross-sectional design prevented a causal interpretation for sleep duration and obesity indicators. Secondly, sleep duration was self-reported. However, the measurement of objective sleep duration needs participants to wear wrist activity monitors which may affect the participants’ sleep [50]. In addition, sleep monitoring can only monitor sleep for one or more nights which may not represent the participants’ habitual sleep duration [49, 51].

## Conclusions

In our study, sleep duration was associated with BMI and VFA, and there were gender differences in this association. Short sleep increased the odds of general obesity and visceral obesity in women, long sleep decreased the odds of visceral obesity in men. However, no association was observed between sleep duration and PBF in either women or men. Gender differences in the relationship between sleep duration and obesity-related indicators are not yet fully clarified, meanwhile the biological mechanisms of this possible gender difference also need further study to explore, more prospective studies with large sample size are needed in the future to confirm the relationship between sleep duration and obesity-related indicators.

## Supplementary information

**Additional file 1.**

**Additional file 2.**

**Additional file 3.**

## Data Availability

The datasets used and/or analysed during the current study are available from the corresponding author on reasonable request.

## Abbreviations

BMI: body mass index
PBF: percentage of body fat
VFA: visceral fat area
PSQI: Pittsburgh Sleep Quality Index
ANOVA: one-way analysis of variance
CT: computed tomography
MRI: magnetic resonance imaging
