# Supplementary material for: Gender differences in the association between sleep duration and body mass index, percentage of body fat and visceral fat area among chinese adults: a cross-sectional study
Source: BMC Endocr Disord. 2021 Dec 24;21:247. doi: 10.1186/s12902-021-00913-4 (PMC8709998; doi:10.1186/s12902-021-00913-4)
Supplement: Supplementary file 2 — Additional file 2. [file 12902_2021_913_MOESM2_ESM.docx]

**Health Assessment Questionnaire**

We are interested in assessing the health status and finding out health related factors, including demographic characteristics, history of chronic diseases, living habits, sleep, etc., among the adult population in the Beijing-Tianjin-Hebei region. Please answer each question truthfully. We guarantee that we will not disclose your personal information and your information will not be used for any purpose other than scientific research in any way under any circumstances. This survey is voluntary and you may refuse to participate or withdraw from it.

**Part 1-Demographic Characteristics**

- 1. When were you born?

□□/□□/□□□□(dd/mm/yyyy)

1-2 What is your current occupation?

□1. Civil servants □2. Professionals □3. Other

1-3 What is your current marriage status?

□1. Unmarried □2. Married □3. Divorced □4. Widowed

1-4 What is your highest education level?

□1. High school or below □2. Bachelor degree □3. Postgraduate and above

**Part 2-** **History of Diseases**

2-1Have you ever been diagnosed with any of the following chronic diseases?

1 Hypertension: □1. Yes □2. No □3. Unknown

2 Diabetes: □1. Yes □2. No □3. Unknown

3 Coronary heart disease: □1. Yes □2. No □3. Unknown

4 Stroke: □1. Yes □2. No □3. Unknown

5 Chronic kidney disease: □1. Yes □2. No □3. Unknown

6 Chronic obstructive pulmonary disease: □1. Yes □2. No □3. Unknown

7 Dyslipidemia: □1. Yes □2. No □3. Unknown

2-2 Have you ever been diagnosed with cancer?

□1. Yes □2. No □3. Unknown

2-3 Have you ever taken any medication regularly for the past three months?

□1. Yes □2. No (Jump to **Part 3**)

2-4 What kind of medications have you been taking regularly in the past three months? □1.lowering uric acid agents □2. anti-arrhythmia agents □3.hormone agents □4. sleeping agents □5.other_______________

**Part 3-Living habits**

3-1 Do you smoke at least once a week for more than 3 consecutive months?

□1. Yes □2. Never □3. Former

3-2 Do you drink at least once a week for more than 3 consecutive months?

□1. Yes □2. Never □3. Former

3-3 Do you have exercise at least once a week for more than 3 consecutive months? (Such as fast walking, running, swimming, yoga, ball game, climbing, Tai Ji)

□1. Yes □2. Never (Jump to **Part 4**)

3-4 How many days do you usually spend on exercise per week?

_____ days per week

3-5 How much time do you usually spend on exercise per day?

_____ minutes per day

**Part 4-Sleep**

4-1 **During the past month**, when have you usually gone to bed at night?

□□:□□(Please fill in the 24-hour schedule)

4-2 **During the past month**, when have you usually gotten up in the morning?

□□:□□(Please fill in the 24-hour schedule)

4-3 **During the past month**, how would you rate your sleep quality overall?

□1. Very good □2. Good □3. Bad □4. Very bad
