# Supplementary material for: Gender differences in the association between sleep duration and body mass index, percentage of body fat and visceral fat area among chinese adults: a cross-sectional study
Source: BMC Endocr Disord. 2021 Dec 24;21:247. doi: 10.1186/s12902-021-00913-4 (PMC8709998; doi:10.1186/s12902-021-00913-4)
Supplement: Supplementary file 3 — Additional file 3. [file 12902_2021_913_MOESM3_ESM.docx]

**Dear Editor,**

**Thank you for the opportunity to revise this paper “Gender differences in the** **association between sleep duration and body mass index, percentage of body fat and visceral fat area among Chinese adults: a cross-sectional study**”. We have responded to the helpful reviewer comments and present a point-by-point response below. This has helped to strengthen the paper and we hope it will now be ready for publication.

Best regards,

Minying on behalf of the coauthors.

**Response to reviewer**

Reviewer reports:

Reviewer 1: I have read the manuscript with much interest. I think the major issues with the study is the cross-sectional design where we have many cohort studies and the use of self-reported data. It is not clear for me what the manuscript add in addition to what we already know. The gender interaction is interesting, but I am not sure the sample size is each sub-category is big enough. I have the following comments:

1.	Please use people first language. For examples adults with obesity not obese adults.

**Response:** We have used “obese adult females” and “obese people” in the manuscript, and we have replaced them with “adult females with obesity” and “people with obesity”.

2.	The introduction is extremely long and lack clear hypothesis. Please reduce the introduction to more than 1.5 pages. The evidence for sleep deprivation as a risk factor for obesity is strong. So the authors can start from this starting point and then explain the data with a focus on Chinese population and the potential impact of gender and then state the hypothesis and aims. Need t include clear information why this study is needed and what it adds to the published literature. Especially that it is cross sectional in nature and has self-reported rather than measures sleep duration

**Response:** We have reduced the introduction section according to the comments.

3.	What are "diet pills"?

**Response:** "diet pills" is the medicine which could help people lose weight.

**Changes:** We have changed it to “weight-loss drugs” to make it clearer.

**(Line 134-136)** Exclusion criteria were: with cognitive impairment, hearing or language impairment, having mental disease, with pacemakers installed, disabled, taking weight-loss drugs.

4.	Was the model adjusted for gender? It does not say that in the methods.

**Response:** Gender was not adjusted in the logistic regression models among the total population because gender-stratified logistic regressions were also conducted.

5.	Why continuous variables were not used in the logistic**?**

**Response:** Several studies indicated that there is not a linear association between sleep duration and BMI. In addition, we have found a U-shaped relationship between sleep duration and BMI which was reported in another manuscript, where sleep duration and BMI were included in the regression model as continuous variables. So the outcome and independent variables were all categorized in the logistic regressions in the current study. With regard to sleep duration, we have referred to a lot of literature where sleep duration was categorized to short, optimal and long sleep duration with different cutoffs. We adopted the cutoffs recommended by the National Sleep Foundation, in which the optimal sleep duration for adults is 7-9 hours per night. However, few participants reported sleeping > 9 h in this study, so we categorized sleep duration to three groups, < 7 h, 7-9 h (including 7 h but not including 9 h) and ≥ 9 h. We categorized PBF, VFA and BMI to obesity and not obesity according to the relevant guidelines.

6.	Interestingly the reported prevalence of short sleep duration is lower than that reported in Western studies; any thoughts why? It will be worth to include in the discussion

**Response:** Compared to Western studies, the reported prevalence of short sleep duration was lower in current study. We speculate the difference may be partly caused by culture difference. It’s reported that sleep duration varies across cultures and countries, thus alter the associations between sleep profiles and disease risks. Therefore, cultural influences on sleep duration and differences in ethnicity across cultures result in diverse relationships between sleep duration and obesity in different countries.

**Changes:** We have added the relevant content to the discussion section.

(**Line 292-295)** We speculate the difference may be partly caused by culture difference. Studies shows that sleep duration varies across cultures and countries, thus alters the associations between sleep profiles and disease risks. Therefore studies on the association between sleep duration and obesity from different countries are needed.

7.	Table 2: Needs the N for each sleep category and for men and women and combined.

**Response:** We have added N for each sleep category, for men, women and the total study population in Table 2.

8.	The authors put a lot of emphasis on the gender differences, but the number of men and women in the short sleep category is about 500 and 700 respectively and that's a small number in sleep duration studies, so the lack of difference in men could simply be lack of power.

**Response:** Though the numbers of men and women reporting short sleep were small in the current study, association between short sleep and general and visceral obesity was still found statistically significant in women, indicating the sample size is large enough and the association is strong enough. However, the small sample size may be one of the reasons why no relationship between short sleep duration and obesity was found among men. Since the study is still in progress, we will expand the study sample size in the future to further explore the association between short sleep and obesity among men.

**Changes:** We have added the related content in the discussion**.**

(**Line 295-298)** Given the lower prevalence of reported short sleep in the current study, the lack of association between short sleep and obesity in men may due to the small number of men reporting short sleep, and research is needed to further explore the association between sleep duration and obesity.

9.	Why doing 2 separate logistic regressions for men and women rather than doing an interaction term?

**Response:** In order to present a clear association between sleep duration and obesity defined by different indicators, we conducted sex stratified logistic regressions rather only including an interaction term in the models. By doing so, we could not only understand the existence of gender-sleep duration interactions in the relationship between sleep duration and obesity, but also clarify the different magnitude and direction of the association of sleep durations with obesity in different sexes.

10.	There is too much repetition between tables and text. No need for the repetition.

**Response:** We have shortened the text in the results section.

11.	Whenever association is mentioned then the direction of this association needs to be clarified and made clear.

**Response:** We have added text to clarify the direction of the association. For example, we added elevated and decreased odds to indicate positive and negative direction of the association (e.g., short sleep was independently associated with elevated odds of general obesity (defined using BMI) and visceral obesity (defined using VFA) among the total study population).

12.	Was there a dose response in the findings?

**Response:** There is not a dose response in the findings. We have conducted another analysis on the association between sleep duration and BMI, [triglyceride](javascript:;) and [cholesterol](javascript:;), where sleep duration and BMI were included in the regression model as continuous variables, and found a U-shaped relationship between sleep duration and BMI which was reported in another manuscript.

13.	When discussing the findings in comparison to other studies, the authors need to provide insights into the differences in study designs, methods of assessing the exposure and outcome measure and differences in populations and sample sizes.

**Response:** We totally agree that differences in study population, designs, sample size, methods of assessing the exposure and outcome measure may all contribute to the different findings on the association between sleep duration and obesity, which have been discussed in the manuscript (e.g., ‘A large Thai cohort of over 60,000 adults reported that short sleep increased the risk of general obesity in both men and women compared with those slept 7 hours[19]. Cross-sectional studies conducted in Spain[35], and Finland[36] showed consistent findings.’)

14.	The discussion needs to be shortened and more focused.

**Response:** We have shortened the discussion and made it more focused.

15.	The mechanistic section is currently weak. This should discuss all possible aspects of energy homeostasis (there are systematic reviews that can be referenced and used to summarise the findings). But the mechanism should also consider the other direction considering the cross-sectional nature of the study.

**Response:** We have presented more detailed mechanisms of the association between sleep duration and energy homeostasis.

**Changes:**

**(Line 328-349)** Although the exact association between sleep duration and obesity-related indicators is not fully clear, several potential mechanisms might explain the link between sleep deprivation and obesity. One is by changing the levels of neurohormones that regulate eating behaviors. Acute sleep deprivation leads to a 28% increase in the average level of appetite-stimulating hormones, while the average level of leptin decreases by 18%. Besides, lack of sleep is associated with increases in hunger, appetite, and hedonic food intake, especially for high-calorie food [42]. Another possible way for insufficient sleep to induce obesity is through activation of the sympathetic nervous system. Insufficient sleep can reduce glucose tolerance, increase cortisol levels and heart rate variability. These neuroendocrine changes interfere with the mechanism of blood glucose regulation, resulting in higher steady-state blood glucose concentration [43]. Additionally, activation of inflammatory pathways may also play a role in the link between short sleep duration and obesity. Studies have confirmed that insufficient sleep is associated with increases in tumor necrosis factor, interleukin 6 and C-reactive protein, which play important roles in obesity[44,45]. Long sleep causes many similar potential metabolic problems, which may differ from that of short sleep [46,47]. Several studies have demonstrated that longer sleep duration among older persons can affect energy metabolism and thereby increase the risk of destroyed energy homeostasis through various possible complex mechanisms, including poor sleep quality, sedentary lifestyle, unhealthy dietary habits, and circadian rhythm [48]. How dose long sleep influence energy homeostasis among young adults is rarely studied yet. However, in addition to the different study population and design, different cutoffs of long sleep were used may result in different findings.

16.	The limitations and strengths is too long. Please list the weaknesses and strengths straight to the point.

**Response:** We have shortened the content on the limitations and strengths in the discussion.
